# Optical Characterization of Sodium Fluorescein *In Vitro* and *Ex Vivo*

**DOI:** 10.3389/fonc.2021.654300

**Published:** 2021-05-10

**Authors:** Ran Xu, Wanda Teich, Florian Frenzel, Katrin Hoffmann, Josefine Radke, Judith Rösler, Katharina Faust, Anne Blank, Susan Brandenburg, Martin Misch, Peter Vajkoczy, Julia Sophie Onken, Ute Resch-Genger

**Affiliations:** ^1^ Department of Neurosurgery, Charité—Universitätsmedizin Berlin, Corporate Member of Freie Universität Berlin, and Humboldt-Universität zu Berlin, and Berlin Institute of Health, Berlin, Germany; ^2^ Division Biophotonics, Federal Institute for Materials Research and Testing (BAM), Berlin, Germany; ^3^ Department of Neuropathology, Charité—Universitätsmedizin Berlin, Corporate Member of Freie Universität Berlin, Humboldt-Universität zu Berlin, and Berlin Institute of Health, Berlin, Germany; ^4^ German Cancer Consortium (DKTK), Heidelberg, Germany, partner site Charité Berlin, Berlin, Germany; ^5^ Berlin Institute of Health (BIH), Berlin, Germany

**Keywords:** sodium fluorescein, spectroscopy, brain tumor, confocal, fluorescein-guided surgery, optical probe, pH sensing, NaFl

## Abstract

**Objective:**

The utilization of fluorescein-guided biopsies and resection has been recently discussed as a suitable strategy to improve and expedite operative techniques for the resection of central nervous system (CNS) tumors. However, little is known about the optical properties of sodium fluorescein (NaFl) in human tumor tissue and their potential impact on *ex vivo* analyses involving fluorescence-based methods.

**Methods:**

Tumor tissue was obtained from a study cohort of an observational study on the utilization of fluorescein-guided biopsy and resection (n=5). The optical properties of fluorescein-stained tissue were compared to the optical features of the dye *in vitro* and in control samples consisting of tumor tissue of high-grade glioma patients (n=3) without intravenous (i.v.) application of NaFl. The dye-exposed tumor tissues were used for optical measurements to confirm the detectability of NaFl emission *ex vivo*. The tissue samples were fixed in 4%PFA, immersed in 30% sucrose, embedded in Tissue-Tek OCT compound, and cut to 10 μm cryosections. Spatially resolved emission spectra from tumor samples were recorded on representative slides with a Confocal Laser Scanning Microscope FV1000 (Olympus GmbH, Hamburg, Germany) upon excitation with λ_exc_ = 488 nm.

**Results:**

Optical measurements of fluorescein in 0.9% sodium chloride (NaCl) under *in vitro* conditions showed an absorption maximum of λ_max abs_ = 479 nm as detected with spectrophotometer Specord 200 and an emission peak at λ_max em_ = 538 nm recorded with the emCCD detection system of a custom-made microscope-based single particle setup using a 500 nm long-pass filter. Further measurements revealed pH- and concentration-dependent emission spectra of NaFl. Under ex vivo conditions, confocal laser scanning microscopy of fluorescein tumor samples revealed a slight bathochromic shift and a broadening of the emission band.

**Conclusion:**

Tumor uptake of NaFl leads to changes in the optical properties – a bathochromic shift and broadening of the emission band – possibly caused by the dye’s high pH sensitivity and concentration-dependent reabsorption acting as an inner filter of the dye’s emission, particularly in the short wavelength region of the emission spectrum where absorption and fluorescence overlap. Understanding the *ex vivo* optical properties of fluorescein is crucial for testing and validating its further applicability as an optical probe for intravital microscopy, immunofluorescence localization studies, and flow cytometry analysis.

## Introduction

The utilization of fluorescein as an optical probe for guiding the resection and biopsy of central nervous system (CNS) tumors has been recently proposed as a powerful strategy to improve and expedite operative techniques (1–4). Sodium fluorescein (NaFl) is a fluorescent dye with a molecular weight of 376.3 g/mol that accumulates in tumor tissue where blood brain barrier (BBB) breakdown occurs and thus, enhances tumor visualization, contributing to the extent of resection which is in turn associated with a better overall survival rate (5–9). This dye has been successfully implemented in clinical applications in ophthalmology; in recent years, its implications for CNS tumor resection, as well as for vascular neurosurgery techniques have also been discussed (10–13).

However, little is known about the spectroscopic characteristics of fluorescein after its intravenous (i.v.) application, as it traverses the BBB and accumulates in tumor tissue. The tumor microenvironment is a complex compartment with spatiotemporal variability in tumor cells, acidity, hypoxia, secreted factors, and extracellular matrix proteins. Moreover, it is unknown how the dye is metabolized in this environment. This encouraged us to characterize the *in vitro* and *ex vivo* optical characteristics of NaFl to understand its spectroscopic features after biological tissue uptake with special emphasis on its potential further implications of fluorescent-based assays.

## Material and Methods

### Patients and Specimen Handling

**Figure 1:**
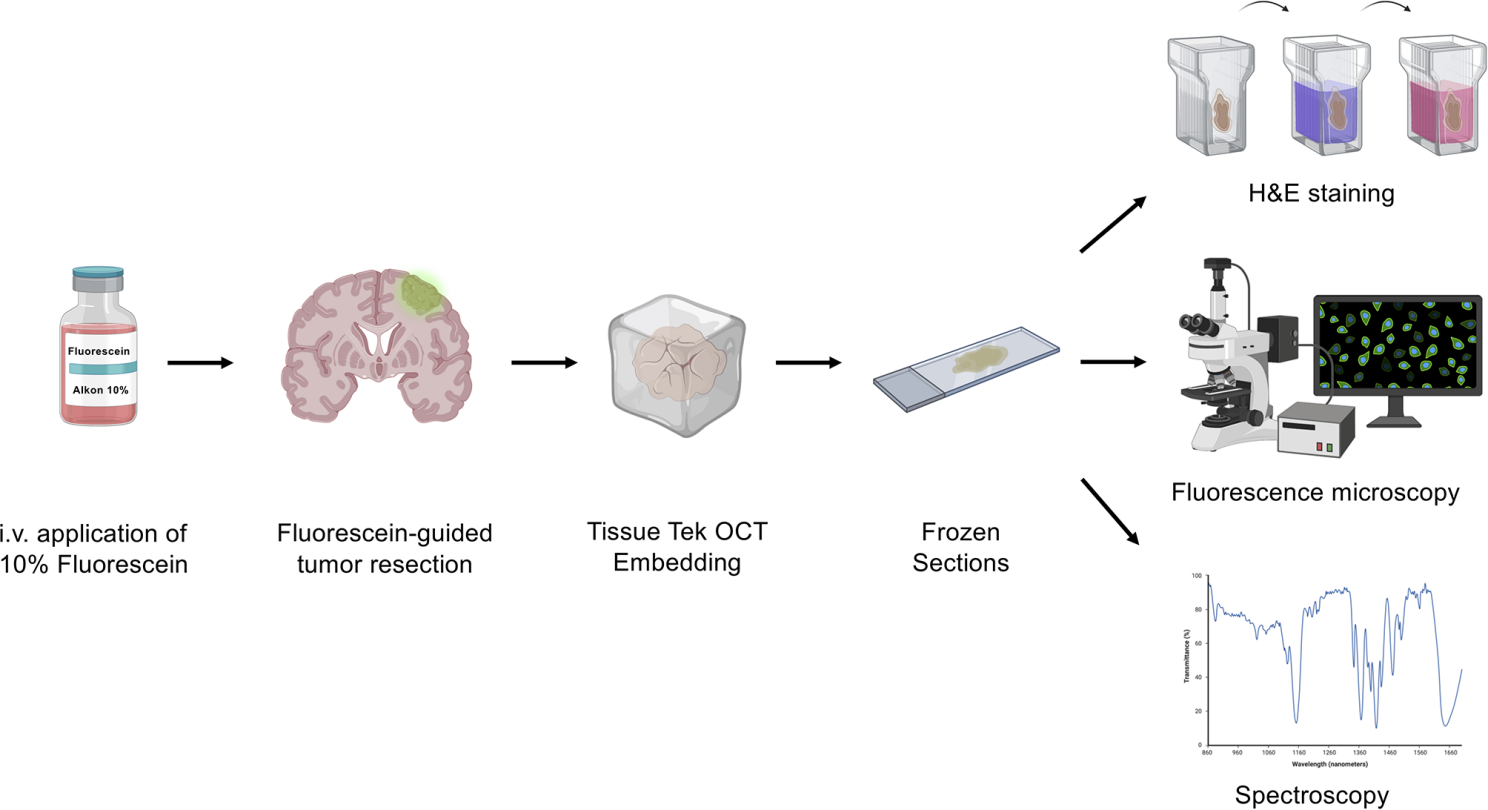
Experimental workflow. Before tumor resection, patients received sodium fluorescein (NaFl) intravenously (i.v.). After fixation in 4% PFA and 30% sucrose immersion, the NaFl-stained tissue was embedded in Tissue-Tek OCT before cryosectioning. Frozen sections were then studied by fluorescence microscopy and spectroscopy measurements.

The study was approved by the local Ethical Committee (EA1/284/20, EA4/219/17 and EA2/101/08) of the Charité University Hospital. All patients gave written consent for the off-label use of i.v. fluorescein-guided surgery or biopsy. All study patients had a contrast-enhancing lesion in which surgical resection was indicated, and their characteristics are listed in **Table 1**. The workflow of the experimental setup of human tissue is shown in **Figure 1**. Briefly, patients received intraoperatively a dosage of 5 mg/kg Fluorescein Alkon i.v. according to our standardized operating procedure for brain tumor surgery (n=5). Tumor surgery was performed in a standard fashion using the Pentero 900 microscope with Y560 filter, Carl Zeiss, Meditec, Oberkochen, Germany. The dye-exposed tumor tissue was brought to the laboratory on ice, and immediately fixed in 4% PFA overnight at 4°C, then immersed in 30% sucrose, and embedded in Tissue-Tek^®^ O.C.T.™ compound. Cryosections were cut into slices of 10 μm thickness. Control samples (n=3) consisted of glioma tumor samples from patients who did not receive fluorescein-guided surgery due to contraindications. Hematoxylin & Eosin (H&E) staining was performed using a standard methodology and staining reagents. Images were collected using a Carl Zeiss Axio observer Z1 inverted immunofluorescence microscope equipped with standard DAPI (filter 49, excitation 365 nm, emission 445 ± 25nm), FITC (filter 38, excitation 470 ± 20nm, emission 525 ± 25nm nm), and Cy3 (filter 43, excitation 545 ± 12.5nm, emission 605 ± 35nm) filters, respectively.

**Table 1:** Patient characteristics.

	Age	Gender	Localization	Extent of resection	Histology
**Fluorescein**	50	M	Left temporolateral	Resection	Glioblastoma, IDH-wildtype (WHO grade IV), MGMT methylated
	58	M	Left temporopolar	Resection	Glioblastoma, IDH-wildtype (WHO grade IV), MGMT methylation
	87	M	Left temporolateral	Biopsy	Glioblastoma, IDH-wildtype (WHO grade IV), MGMT unmethylated
	71	F	Left temporomesial	Biopsy	Diffuse large B-cell lymphoma
	61	M	Left occipital	Resection	Post-transplant lymphoproliferative disorder (PTLD)
**Control**	24	F	Left temporomesial	Resection	Glioblastoma (WHO grade IV), MGMT unmethylated
	35	F	Right temporolateral	Resection	Glioblastoma (WHO grade IV), MGMT methylation
	64	F	Left frontal operculum	Resection	Glioblastoma (WHO grade IV), MGMT methylation

F, female; M, male.

### Spectroscopy of NaFl *In Vitro*

The absorbance spectrum of fluorescein in 0.9% NaCl was measured in a cuvette with the spectrophotometer Specord 200. The emission spectrum of fluorescein in 0.9% NaCl under 488 nm laser excitation (Supercontinuum Laser Solea, PicoQuant) was recorded with a custom-made microscope-based single particle setup equipped with a 500 nm long-pass filter using the emCCD detection system (DU970P-BVF, Andor Ltd.).

Concentration- and pH-dependent emission spectra of NaFl *in vitro* were recorded using a spectral scanning Confocal Laser Scanning Microscope (CLSM) FluoView FV1000 (Olympus GmbH, Hamburg, Germany). The emission spectra were measured in droplets of the corresponding NaFl solutions, which were placed on standard microscopy coverslips #1.5 (0.170 mm). A multiline argon ion laser (30 mW) was used as excitation source (λ_exc_ = 488 nm), which was reflected by a beam splitter (BS 20/80) and focused onto the sample through an Olympus objective UPLSAPO 20x (numerical aperture N.A. 0.75). The emitted photons were collected with the same objective, focused onto a photomultiplier (PMT), and recorded in the wavelength range of 500 - 640 nm (spectral resolution of 2.0 nm, step sizes of 2.0 nm).

### Spectroscopy of NaFl *Ex Vivo*

Spatially resolved emission spectra of tumor samples were recorded on representative tumor sections. Exemplarily chosen, but representative 10 regions of interests (ROIs) within the tumor tissue and one additional ROI as a blank or baseline control (recording background noise) were measured with the FV1000 (Olympus GmbH, Hamburg, Germany) upon excitation with λ_exc_ = 488 nm under almost the same experimental settings and conditions as described above. The *ex vivo* spectra, however, were measured in derogation through an objective UPLSAPO 20xW/0.75 in the range of 500 nm - 740 nm (spectral resolution of 5.0 nm and step sizes of 2.0 nm).

### Statistical Analysis and Figures

Values are generally presented as mean +/- SEM unless otherwise stated. Statistical significance was determined by Student t-test and statistical analysis was performed using Graphpad Prism software (Version 7.0). Elements of **Figures 1** and **5** were composed using BioRender.Com.

## Results

### NaFl Tumor Samples Show Positive Signal in Both Green and Red Channels

**Figure 2:**
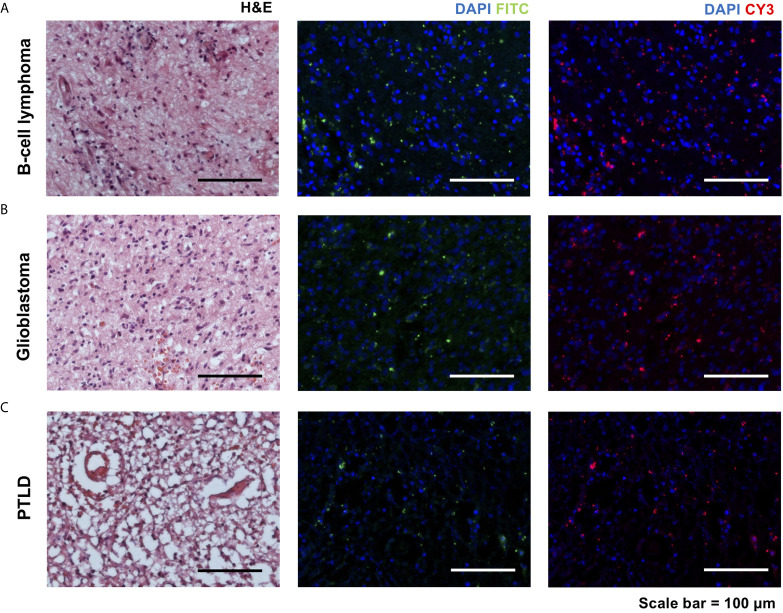
Hematoxylin and eosin (H&E) staining of rapid sections in three different pathologies [**A**, B-cell lymphoma, **B**, glioblastoma, and **C**, posttransplant lymphoproliferative disorder (PTLD)] with consecutive slides using FITC and CY3 filters of an immunofluorescence microscope. All samples showed signals in the FITC channel, and unexpectedly, also in the CY3 channel (scale bar = 100 µm).

To examine whether NaFl tumor tissue still showed measurable FITC signal after fixation, frozen sections were analyzed in the FITC channel of a fluorescent microscope. Indeed, all the samples exhibited a heterogeneous pattern of FITC signal (**Figure 2**). To clarify that the area of interest was in fact vital tumor tissue, consecutive sections were subjected to Hematoxylin & Eosin (H&E) staining and examined by a consultant neuropathologist to confirm the histopathological diagnosis of the rapid frozen sections (**Figure 2**). However, all samples showed also fluorescence signals in the CY3 channel (**Figure 2**). This observation was surprising since NaFl should not reveal a signal in the CY3 channel separated by the CY3 filter (covering the emission wavelength region of 605 ± 35nm by its transmission profile) under baseline *ex vivo* conditions. The observed changes in the spectroscopic features of NaFl might be of significant relevance in the future, e.g. for the in-depth analysis of *in vivo* tumor imaging using NaFl. To better understand this shift in emission wavelength we performed a series of *in vitro* and *ex vivo* studies to characterize the optical properties of our optical probe.

### Absorbance and Emission of NaFl *In Vitro*

**Figure 3:**
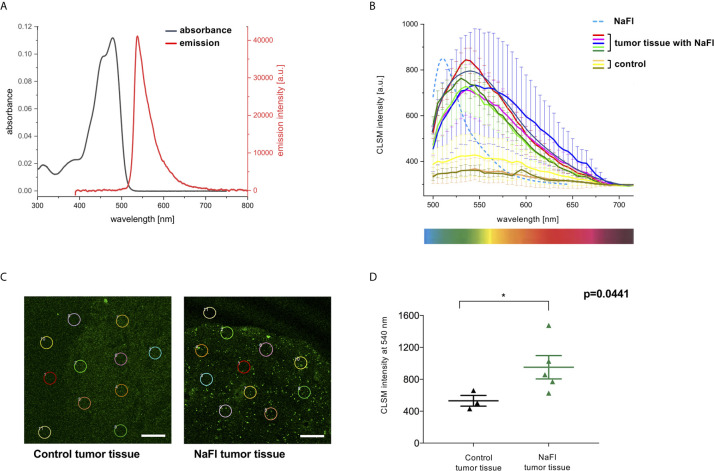
Absorbance and emission of NaFl *in vitro* and *ex vivo*. **(A)** Absorbance (black line) and emission spectra (red line) under *in vitro* conditions show the typical maximum absorbance at λ_max abs_ = 479 nm and an emission peak at λ_max em_ = 538 nm. **(B)** Confocal Laser Scanning Microscopy (CLSM) emission spectra of NaFl in phosphate buffer solution at pH 8.04 *in vitro* (blue dashed line) and in *ex vivo* samples (colored solid lines). Exemplary, but representative *ex vivo* tumor samples with NaFl show a broadened, bathochromically shifted NaFl tumor emission (solid lines). A broad but very weak emission in the same wavelength region was also observed for the control samples. **(C, D)** Exemplary, but representative CLSM intensities of 10 regions of interests (ROIs) read out at 540 nm show a significantly higher signal in fluorescein tumor samples (971.1 ± 146.1 a.u.) versus control samples (530.6 ± 67.58 a.u.); p= 0.0441. Values are expressed in average ± S.E.M. (Scale bar = 100 µm).

Next, to elucidate the optical properties of NaFl *in vitro*, we measured absorbance and emission spectra of NaFl under *in vitro* conditions. Here, we observed a typical absorbance peak at λ_max abs _= 479 nm and an emission peak at λ_max em _= 538 nm (**Figure 3A**). These absorbance and emission maxima match well with the previously reported spectroscopic features of fluorescein (14).

### Fluorescein-Stained Tumor Tissue Shows a Bathochromic Shift in Emission *Ex Vivo*

We next compared the *in vitro* emission spectra of NaFl to the corresponding emission spectra obtained *ex vivo* to better understand the optical behavior of this dye within the tumor core. Interestingly, in representative tumor samples, we observed a broadening of the dye’s emission band and a slight, but significant spectral shift to longer wavelengths, also known as a bathochromic shift, compared to NaFl in buffer solution pH 8.04 *in vitro* (**Figure 3B**). The control samples showed a significantly lower emission intensity with a mean intensity of 530.6 (± 67.58) a.u. [= arbitrary (or relative) units] compared to 971.1 (± 146.1) a.u. at a wavelength of 540 nm in the NaFl tumor samples (p=0.0441) (**Figures 3C, D**).

### pH-Dependent and Concentration-Dependent Emission

**Figure 4:**
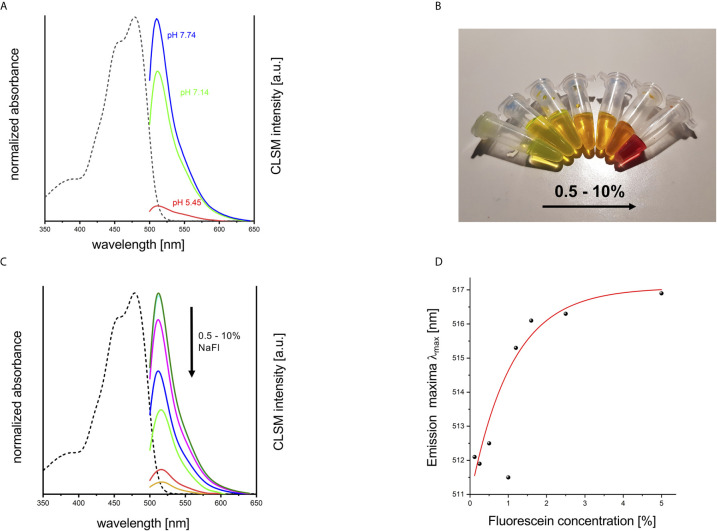
pH- and concentration-dependent emission of NaFl. **(A)** Normalized absorbance (in 0.9% NaCl solution; dashed line) and pH dependent emission spectra of NaFl solutions in different phosphate buffers (phosphate buffer pH 5.54, pH 7.35, and pH 8.04) obtained with a confocal laser scanning microscope (CLSM). **(B)** Concentration series of Na-Fl solutions (in 0.9% NaCl), and **(C)** the corresponding absorbance (in 0.9% NaCl solution; dashed line), and concentration-dependent CLSM emission spectra (solid lines). **(D)** Emission maxima λ_max_ [nm] plotted against NaFl concentration [%] show a concentration-dependent red shift by about 5 nm.

Since NaFl showed heterogeneous uptake in tumor tissue, we examined whether this finding could be explained by the characteristic pH-dependent emission features of the dye - also given that previous studies highlighted an acidic milieu of the tumor microenvironment contributing to a change in pH (15, 16). Therefore, we measured the absorbance spectra of NaFl in 0.9% NaCl solution and its emission spectra in different phosphate buffers (pH 5.54, pH 7.35, and pH 8.04). Our results reveal the well-known pH-dependence of the NaFl emission intensity that increases with increasing pH (**Figure 4A**) (17). Also, the heterogeneous uptake of the dye leading to different local NaFl concentrations could contribute to the spectroscopic effects observed *ex vivo*. Hence, we measured the spectra of NaFl *in vitro* at different dye concentrations. Thereby, we noticed not only a concentration-dependent emission intensity of the dye (**Figures 4B, C**), but also observed a red shift in the emission maxima with increasing NaFl concentrations (**Figure 4D**).

## Discussion

**Figure 5:**
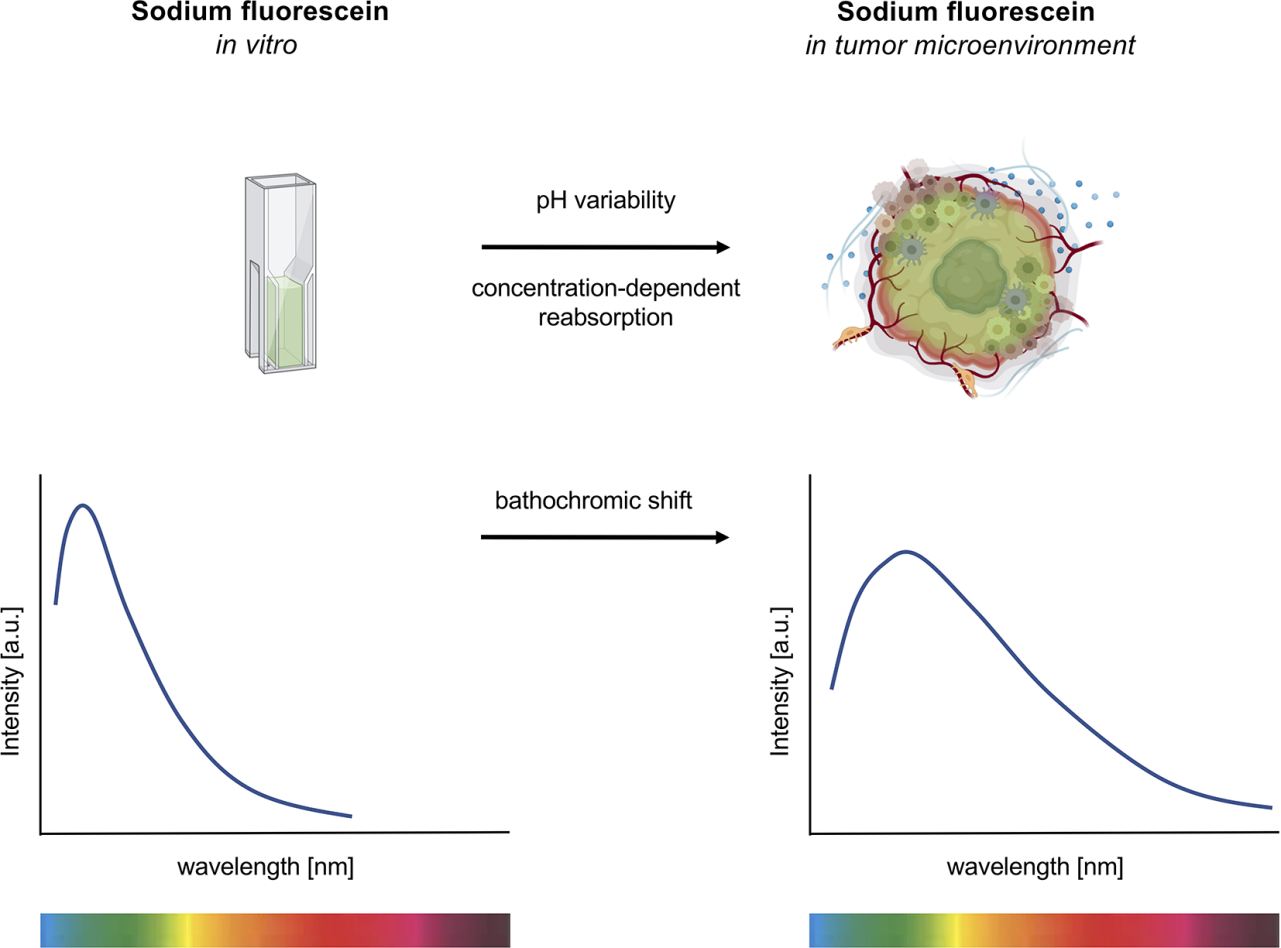
Emission spectroscopy of sodium fluorescein (NaFl) under *in vitro* and *ex vivo* conditions. Exposure of NaFl to the tumor microenvironment shows a broadening and a bathochromic shift of the dye’s emission spectrum. The pH sensitivity and the concentration-dependent reabsorption effect of the dye contribute to this red shift.

Our results show that NaFl exhibits a significant broadening of its emission band together with a bathochromic shift after tissue uptake in CNS tumor samples. These changes could possibly be explained by the dye’s high pH sensitivity and/or concentration-dependent reabsorption effects (**Figure 5**).

Furthermore, our data reveal a very heterogeneous distribution pattern of NaFl in tumor tissue on a microscopic level. This can also be observed intraoperatively by the neurosurgeon on a macroscopic scale. Traditionally, a heterogeneous uptake can be related to a variability in BBB properties and regionally distinct hyperpermeability. Our data show that this heterogeneous emission signal of the dye is also influenced by the dye’s high pH sensitivity, potentially caused by the variability of the acidic milieu in the tumor microenvironment. This acidic milieu has also been discussed in the literature as a driver of cancer development (15). The fluorescein molecule can exist in different protonation states which all differ in their fluorescence features; only the dianion and monoanion of fluorescein emit a visible fluorescence of varying fluorescence efficiency or quantum yield while the neutral dye is non-emissive (17). A more acidic extracellular milieu should lead to the formation of the monoanion while a more basic environment favors the formation of the dye’s dianion. This goes along with the pH-dependent emission spectra found for the dye in different buffer solutions. Moreover, the heterogeneous appearance of NaFl uptake in tumor tissue is likely caused by the high sensitivity of the NaFl emission behavior to its concentration. This corresponds with the observed decrease in fluorescence intensity with higher local dye concentration due to reabsorption effects. Our data confirm earlier reports that both the pH of the dye’s local environment as well as dye concentration can affect the emission of NaFl, which could explain the findings of our *ex vivo* optical measurements, showing a red shift in fluorescence (18, 19).

The change in the intrinsic spectroscopic properties of fluorophores including spectral shifts and changes in the shape of fluorescence emission spectra caused by different environments have been reported before, and the tumor microenvironment is a complex system with a considerable variability in tumor cells, acidity, secretomes, and extracellular compartments that can likely cause changes in the dye’s spectroscopic features (4). For example, another study also reported a bathochromic shift for fluorescein ligands bound to rabbit polyclonal anti-Fluorescein Fab fragments (20).

Tumor tissue autofluorescence is a common phenomenon that should be considered, specifically when working with fluorescence-based assays. Some studies even suggest to utilize the spectral characteristics of the autofluorescence signals from glioma cells for diagnostic purposes (21, 22). To verify that autofluorescence signals do not potentially resemble or interfere with the NaFl signal, we measured the emission spectra of native tumor tissue without exposure to NaFl under the same conditions as used for the NaFl measurements. Although this experiment revealed a broad emission band in the green and red wavelength region, control tumor tissue showed only a very weak emission signal compared to the NaFl-stained tumor samples.

Since NaFl has been increasingly used for the guidance of brain tumor resection, it is crucial to understand its *ex vivo* optical properties, since they can impact further in-depth *in vivo* imaging analyses as well as fluorescence-based assays such as FACS analyses, immunofluorescence staining or *in vivo* microscopy (23, 24). The observation in our study that NaFl in tumor tissue revealed signals in the longer wavelength CY3 channel was surprising. The practical implication of these spectral changes is that if further fluorescence-based laboratory studies are conducted on CNS tumor tissue which were resected under fluorescein-guidance, labeling with secondary antibodies using dyes emitting in the FITC or Cy3 channel should be used with care as this can result in spectral interferences and crosstalk. Based on the broadening and bathochromic shift of the emission band, it seems to be more reasonable to switch to secondary antibodies labeled with dyes emitting in the blue (e.g. 350 – 408 nm) or preferably in the far red (630 – 647 nm) and near-IR channels (650 – 750 nm) in assays such as immunofluorescence microscopy and flow cytometry. The implications of the observed changes in optical properties are of relevant nature, especially if these fluorescent-based assays are used for further diagnostic purposes. Furthermore, in the clinical setting, the utilization of NaFl in combination with in-depth *in vivo* and *ex vivo* imaging techniques such as confocal laser endomicroscopy has also been discussed in recent studies in aiding histopathological diagnoses (25, 26). Hence, the increasing role of NaFl in such settings poses increasing interest on NaFl tumor kinetics, and further analysis of its spectroscopic features may help to distinguish its free form from fluorescein metabolites (fluorescein glucuronide).

In summary, our data reveal changes of the spectroscopic properties of NaFl *ex vivo*, particularly a bathochromic shift in emission after tumor uptake, underpinning that the widespread use of fluorescein in neurosurgical procedures requires a detailed study of its optical properties in a clinical setting. Our study is, however, limited by a small sample size, heterogeneity in tumor histology, and the absence of *in vivo* pharmacokinetic properties of NaFl. Further studies are needed to fully understand the exact nature of the changes of the spectroscopic properties of NaFl *ex vivo* and related possible interference with other fluorescence-based assays in tumor tissue.

## Data Availability Statement

The raw data supporting the conclusions of this article will be made available by the authors, without undue reservation.

## Ethics Statement

The studies involving human participants were reviewed and approved by Charité’s Ethics Committee. The patients/participants provided their written informed consent to participate in this study.

## Author Contributions

WT, KH, FF, JoR, AB, SB, and RX conducted experiments and analyzed data. RX, JO, and UR-G designed the study. All authors contributed to writing and revising of the manuscript. All authors contributed to the article and approved the submitted version.

## Funding

RX is supported by the BIH-Charité Clinician Scientist Program funded by the Charité—Universitätsmedizin Berlin and the Berlin Institute of Health. We acknowledge support from the German Research Foundation (DFG) and the Open Access Publication Fund of Charité—Universitaétsmedizin Berlin.

## Conflict of Interest

The authors declare that the research was conducted in the absence of any commercial or financial relationships that could be construed as a potential conflict of interest.

The reviewer JG declared a past co-authorship with the authors to the handling editor.
